# Supplementary material for: Complexome profiling of the Chlamydomonas psb28 mutant reveals TEF5 as an early PSII assembly factor
Source: Plant Cell. 2025 Mar 17;37(6):koaf055. doi: 10.1093/plcell/koaf055 (PMC12164590; doi:10.1093/plcell/koaf055)
Supplement: koaf055_Supplementary_Data [file koaf055_supplementary_data.zip › psb28_interactive.zip!/plots/intensity/qualityPlots/peptides/FAP24_Cre02.g081050.t1.2.html]

### Cre02.g081050.t1.2

Trivial name: FAP24  
SeqCoverage [median]: 42.50  
Mass: 55.89 kDa  
  
 

| Sequence | MissedCleavage | Mass [Da] | wtIntensity | muIntensity | Score |
| --- | --- | --- | --- | --- | --- |
| ALYGLTDAAALTDLK | 0 | 1534.82 | 64729.51 | 189717.48 | 304.57 |
| APEQVVLYFNAYR | 0 | 1568.80 | 6998.60 | 9147.15 | 211.37 |
| APEQVVLYFNAYRR | 1 | 1724.90 | 1801.40 | 34926.99 | 111.79 |
| EAAANTAFNAIVSAYNANK | 0 | 1938.94 | 24247.86 | 31626.63 | 212.92 |
| FLANQR | 0 | 747.40 | 107676.65 | 169297.86 | 155.40 |
| FVGFSVKPSDPQAITFTATADPGPLNR | 1 | 2832.44 | 18508.91 | 0.00 | 122.79 |
| ILNVLDR | 0 | 841.50 | 145921.20 | 530266.32 | 163.34 |
| NMFASNPGR | 0 | 992.45 | 94187.52 | 164935.76 | 184.04 |
| NPTQITLDDFR | 0 | 1318.65 | 4953.04 | 26000.41 | 182.64 |
| NSAVSPLVAWIYYQPK | 0 | 1834.96 | 906.46 | 0.00 | 66.25 |
| PSDPQAITFTATADPGPLNR | 0 | 2068.02 | 0.00 | 1124.70 | 39.82 |
| QISSSSVQFLQDR | 0 | 1493.75 | 94209.63 | 253885.94 | 299.09 |
| QLAATYLSK | 0 | 993.55 | 98833.46 | 100900.38 | 173.48 |
| QYVQDAGGR | 0 | 992.47 | 53334.90 | 57335.52 | 203.02 |
| RQYVQDAGGR | 1 | 1148.57 | 2388.02 | 2473.43 | 110.53 |
| TSDNAVADK | 0 | 919.42 | 4453.12 | 8202.32 | 200.56 |
| TSDNAVADKIDGK | 1 | 1332.65 | 295443.19 | 326422.82 | 180.30 |
| VLAVPLTAVSVQDTTVLNFMK | 0 | 2245.24 | 1373.48 | 0.00 | 92.99 |
| VTGVNVFPTTR | 0 | 1189.65 | 156632.13 | 443597.37 | 250.16 |
| VVTNLYTFAK | 0 | 1154.63 | 78143.80 | 230113.40 | 196.52 |
| YDSATYK | 0 | 846.38 | 44289.97 | 74691.69 | 148.98 |
| YDSATYKAPEQVVLYFNAYR | 1 | 2397.16 | 2431.42 | 0.00 | 104.92 |
| YVATFFNR | 0 | 1016.51 | 110154.64 | 324483.42 | 229.64 |
|  |  |  | 1411618.9 | 2979149.6 |  |
